# Constructing a three-dimensional graphene structure via bonding layers by ion beam irradiation

**DOI:** 10.1038/s41598-019-44697-z

**Published:** 2019-05-31

**Authors:** Mohammad Ali Abdol, Sadegh Sadeghzadeh, Maisam Jalaly, Mohammad Mahdi Khatibi

**Affiliations:** 1 MSc Student of Nano Technology, School of new technologies, Iran University of Science and Technology, Tehran, Iran; 2 Assistant Professor, School of New Technologies, Iran University of Science and Technology, Tehran, Iran; 3 Assistant Professor, School of New Technologies, Iran University of Science and Technology, Tehran, Iran; 4 Assistant Professor, Mechanical Engineering department, Semnan University, Semnan, Iran

**Keywords:** Mechanical and structural properties and devices, Nanopores

## Abstract

In recent years, the use of the multilayer graphene sheets has been considered more than the single-layer due to the cost-effectiveness and the possibility of mass production. But this type of graphene has some kind of structural weakness due to the weak physical link between its layers. Then, in order to strengthen, many structural modifications are proposed by various techniques to manage the mechanisms at interlayer distances. In this study, the focused ion beam irradiation method has been examined to cross-link and strengthen multi-layer graphene sheets with the help of the molecular dynamics simulation technique. Then, uniaxial and transverse tensile tests were performed to check the mechanical properties of obtained cross-linked multilayer graphene sheets. The results of this research can be considered for the creation of a new class of graphene structures. Such structures could be implemented as a membrane in water desalination or as a storage foam in hydrogen or carbon dioxide storage.

## Introduction

The increasing population and global concerns for safe water supply, the global warming due to the burning of non-renewable energy sources and the scarce resources from fossil fuels, all have made water and energy two of the most challenging issues of the present century. In order to overcome the leading environmental crises, there has been an increasing effort to discovery, design and invent more advanced materials to store renewable energy and provide potable water. In the last decade, graphene and its derivative structures have attracted the attention of researchers in various fields of science, due to their exceptional properties. Graphene-related studies are not unique to the single layer of this material and include a wide range of multilayered, composite, and hybrid structures of this material. One of the many types of these structures, which has a lot of potential in many fields, is multi-layered graphene with transverse bonds. In this unique material, the existence of transverse covalent bonds, in addition to strengthening the structure of graphene, is a three-dimensional structure with high porosity and high specific area. Until now, several factors have been proposed to create graphene framework structures, such as boronic acids^1^, diamine monomers^2^, aldehydes^3^, polypropylene imine^4^, iodobenzene^5^ and diisocyanates^6^. With their placement in the graphene sheets and the connection with a surfactant, three-dimensional structures with intermediate spaces and a high surface area could be constructed. Recently, researchers have succeeded in controlling the intermediate space of graphene layers and filling the distance with epoxy and completely fixing it without using the columnar agent and only by controlling the moisture content. This controlled intermediate space between the graphene plates can selectively pass through water molecules and prevent the transfer of ions in the water^7^. Such a structure is an ideal membrane in the field of desalination and water purification. In the meantime, we see theoretical structures that have not been synthesized yet, such as pillared graphene, which was introduced by Dimitrakakis *et al*., in 2008^8^, a structure consisting of single layers of graphene bonded by carbon nanotubes. By the simulations, it has been proven that the doped type of this material with lithium cations can store an appropriate amount of hydrogen that has been determined for applied uses under environmental conditions. Researchers are working to create such structures to transform the energy storage process in the near future.

One of the techniques that can be used to connect graphene sheets is the irradiation technique, which has already been applied in theory and in practice to connect carbon nanotubes^9–11^. In 2014, Wu *et al*., In a study conducted by the molecular dynamics simulation technique, introduced a method called “Nano joining of graphene sheets by ion beam irradiation”. They showed that two overlapping graphene sheets can connect with the carbon ion beam. The two mechanisms observed for this connection, have been introduced the formation of coordination deflection and the trapping of ions between the plates. Among the most influential parameters on the binding properties, are beam energy and irradiation dose. Optimizing theses can results in better mechanical properties of the binding region than pure graphene. The optimum energy level and the dose of the beam found to be 40 eV and 1.06 × 10^15^ cm^2^ respectively^12^. In a similar simulation, argon beam is also used to joining graphene sheets. In this study the mechanism has been proposed as a main factor for the connection, is the creation of dangling bonds during the irradiation process. The tendency of these bonds to saturate and atomic reorganization in the area of the defect causes the formation of the covalent bonds between the sheets. It has been proven that the temperature increase is a beneficial factor that accelerates the saturation process and develops the process of reorganization. In addition, in order to emphasize on the role of irradiation, another experiment was conducted in which the possibility of graphene joining has been studied by applying a large force above the sheets instead of irradiation, and it has been shown that without the irradiation it isn’t possible to join the graphene sheets. In the end, the graphene plane’s chirality effect on the bonding process has been studied, and it has been determined that the plane with the same chirality result in a better connection^13^. In another study, the effect of focused ion beam irradiation on the graphene stack has also been investigated. It has been observed that a focused ion beam can form covalent bonds between graphene sheets in the stack. In the case of the mechanism of the phenomenon, it is stated that under the irradiation process, large amounts of carbon atoms are removed from the surface of the first layer. By continuing the process of irradiation, carbon atoms with dangling bonds brought around the nanopore, these carbon atoms tend to saturated with other carbon atoms. Thus, the saturation of carbon atoms with dangling bonds in different layers leads to the formation of cross-link between different layers^14^. In 2017, Wu *et al*. tried to actually test the joining of graphene sheets by ion irradiation. they used 40 eV of nitrogen ion for this purpose, and then used Raman spectroscopy characterization and atomic force microscopy to test the bonding of the layers, the results indicate the probability of connecting the pages in the overlap area^15^.

In this study, by means of molecular dynamics simulation and exploiting the idea of bonding the layers by the irradiation process and the controlling of the distance between the graphene layers by moisture content, a method for constructing three-dimensional structures of graphene using focused ion beam irradiation proposed. The irradiation of the graphene structure in addition to creating bonds between the individual planes, also can cause the connection between graphene stacks in the border regions. The strength of this three-dimensional structure perpendicular to the graphene sheets is calculated with a range from about 4 to 6 GPa, with respect to the diameter of the beam, which is significant in comparison with other substances. It seems that the use of this technique along with the method of controlling the distance between graphene layers by moisture content, pillared structures of graphene can be obtained with an appropriate strength, which can have many applications in the fields of water and energy. This structure is a suitable candidate for use in membrane filtration systems for water^16^, storage and absorption of gas such as hydrogen and carbon dioxide^17–20^, lithium-ion batteries^21^, catalytic processes^22^ and adsorbents^23^.

## Materials and Methods

All simulations of this study were done with the help of large-scale atomic/molecular massively parallel simulator (LAMMPS) software package^24^. Also, all images were made by Ovito software^25^, and all manipulations and modifications to the structures were done with this software too. The graphene building was created with the commands related to the custom lattice. The graphene lattice was defined in such a way that the distance between the carbon atoms in the graphene honeycomb structure is equal to 1.42 Å^26^. The distance between the planes in the base state of the multi-layered graphene structure was 3.4 Å, and the graphene plane dimensions in all simulations were 3 × 3 nm.

In the ion beam irradiation simulation, the silicon element was selected as an ion particle^27^. The energy of the collision particle was set to 40 eV^12^. Due to the high kinetics of the system and preventing system failure and explosion, the system time step was set to 0.1 fs. Due to the excessive temperature rise in the system during ion irradiation, graphene temperature was set up by a nose hoover thermostat algorithm in NVT ensemble at 300 K and the temperature damping parameter was 100 times of the time step. The microcanonical ensemble (NVE) was used for ion beam, otherwise, the velocity of the particles would be overshadowed and diminished. The potential used for this simulation is a hybrid potential of CH-Airebo^28^ and Tersoff-ZBL^29^ potentials. The Airebo potential models the formation and failure of covalent bonds between carbon atoms in the graphene structure, and the Tersoff-ZBL potential also models the interaction between silicon ions and graphene atoms^27^. The angle of the collision is 90 degrees to the graphene plane and it is perpendicular to the sheets.

**Figure 1:**
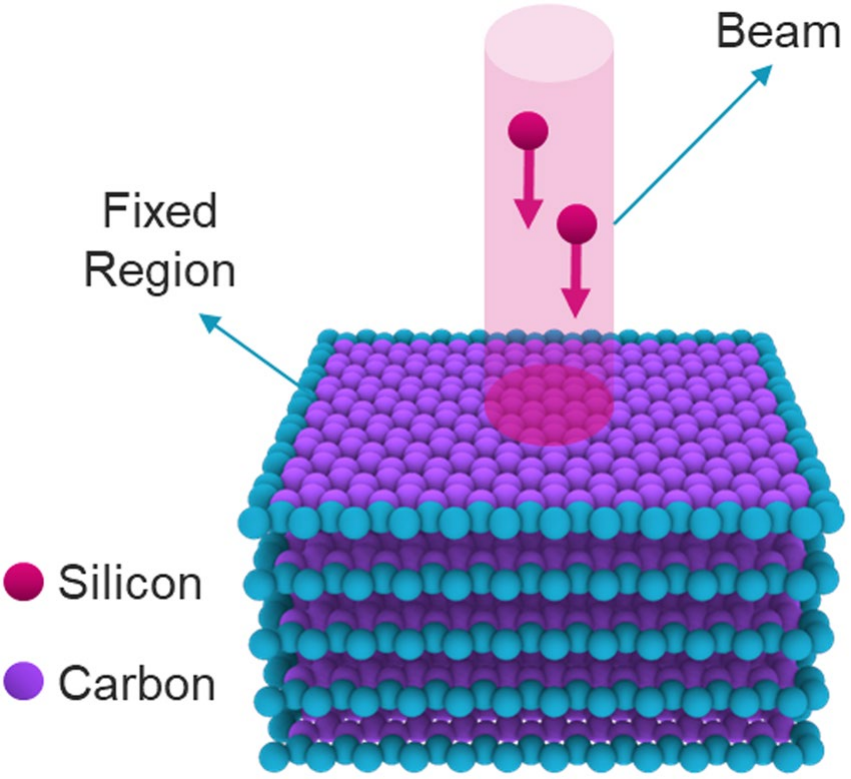
A schematic image of the irradiation process on the graphene layers stack.

Atoms around the layers were fixed to prevent the movement of the layers and the collapse of the initial spacing of the planes during the irradiation process. Applied ensemble to the fixed atoms was NVE. In (Fig. 1), a schematic illustration of the simulation is shown.

The total time of the simulation process was 1.4 million time steps. Prior to the ion collision, graphene was placed at a temperature of 300 K for 200,000 time steps or 20 picoseconds in order to stabilize the original structure in the NVT ensemble. The total amount of time that graphene exposed to the beam was 100 ps. The total dose of collision ions was considered to be 1,000 to ensure the formation and completion of the pore within the graphene structure. Also, between every two collisions, 1000 time steps were taken to give the system an opportunity to be stabled. Finally, after completing the collision process, before exporting the data from the configuration, the graphene structure for the next 20 ps was placed in the NVT ensemble to stabilize, then the configuration data file was extracted for loading in tensile mechanical testing. The irradiation simulation according to the nature of the random collisions of particles was repeated for 5 times, in order to increase the accuracy of the measurements in the same conditions, in the mechanical test by averaging the data.

To investigate the mechanical properties of the structure, the strain-stress test was carried out in two directions, perpendicular to the graphene sheets in the direction of the z-vector and parallel to the graphene sheets along the x-vector. The potential used in stress and strain test is Airebo potential. In various researches, in order to correct the potential, the amount of minimum cut-off radius has been modified^26,30–34^. Here also, this value is changed from 1.72 to 2 Å. It was observed that the selection of values other than 2 Å results in discrepancies in the results and plots of strain-stress testing in different time steps. By correction of the minimum cut-off radius, the problem of non-conformance in the data was solved. The time step for mechanical simulation was selected to 0.1 fs. The strain rate in order to accelerate the simulation process was selected to 0.01/ps. The high strain rate causes a difference of less than 5% in the final graph, but the simulation speed increases by about ten times. In all strain-stress test simulations, before starting the stretching process, the structure in order to stabilize placed in an NVT ensemble at a temperature of 300 K for 20 ps. The output data extraction rate was every 1000 time steps. Also, as mentioned earlier, each of the graphs is the result of averaging of five tests in completely identical conditions to ensure the accuracy of the results. In the stress calculations, due to the irregular geometry created after the irradiation process, the atomic volume of carbon (8.789 Å^3^) and its multiplication in the number of atoms are used to compute the total system volume.

## Results and Discussions

To investigate the effect of irradiation on a multi-layered graphene sheet, 18 different states were investigated, which has been expressed by three main parameters. These three main parameters are: beam energy, beam diameter and the distance between the graphene layers. For the ion beam, two energy levels of 40 eV and 160 eV were considered to study the effect of increasing energy or velocity of particles, on the formation of covalent bonds between graphene layers. The next parameter is the diameter of the beam, for this quantity three different sizes were considered: 0.5 nm, 1.0 nm, and 1.5 nm. The last parameter is the distance between the graphene layers. This distance also has three sizes of 0.34 nm, 0.65 nm and 0.90 nm.

An overview of the results shows that the increase in the beam energy from 40 to 160 electron volts has no considerable effect on the final strengths. Increasing the beam diameter from 0.5 to 1.5 nm and the gap from 0.34 to 0.65 increases the strength from 50 to 100%, respectively. Increasing the gap from 0.34 and 0.9 nm, for all three sizes of pores, reduces the strength of about 33%. Details are presented in the following sections.

**Figure 2:**
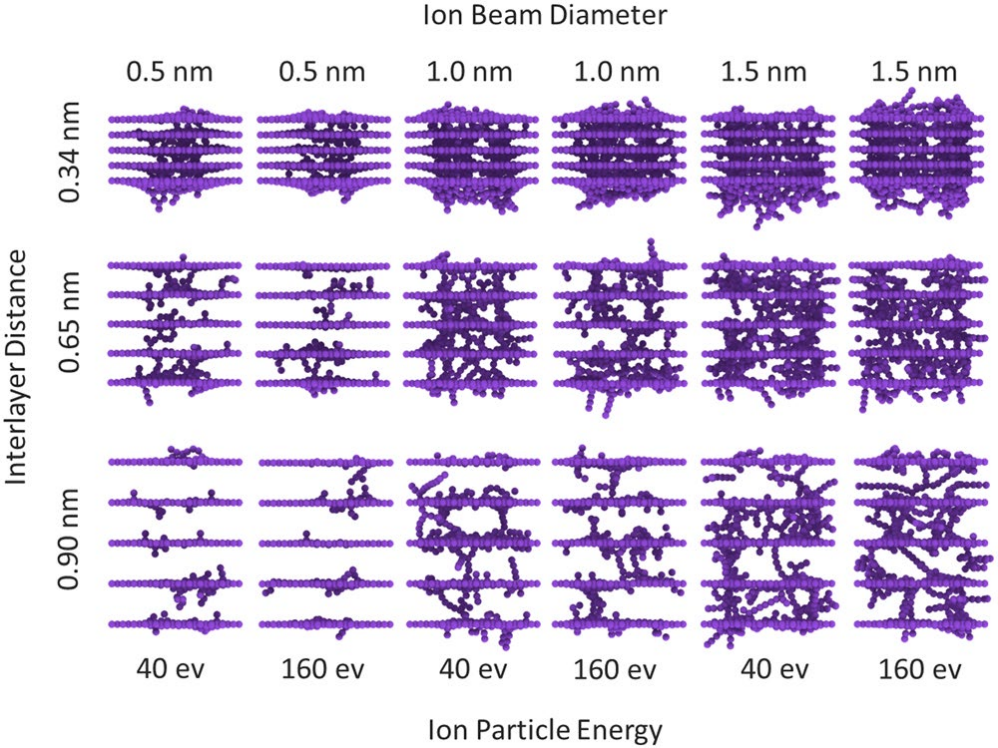
Image of all configurations created after the irradiation process on five-layer stacks of the graphene sheet. The upper axis represents the diameter of the beam, the left axis shows the distance between the layers in the stack and the lower axis indicates the energy of the used beam.

In Fig. 2, all of the 18 structures created after the irradiation process are compared with each other. According to the output images, it appears that between three expressed parameters, the first one, namely beam energy, have the least effect on the final structure, and the difference between the two structures created by different levels of energy is not tangible. In the case of the second parameter, or the diameter of the beam, an increase in the diameter from 0.5 nm to 1.5 nm has a significant effect on the increase in the volume of the intermediate links between the graphene sheets. In structures with a small collision diameter, there is no bond between layers. Also, in medium and large diameters, there are layers that are bond-free. And with the increase of the third parameter, the distance between the layers, we see that the density of the bonds is reduced, and short bonds have been converted to long chains between graphene layers. In brief, by increasing the size of the diameter of the beam, the number of covalent bonds increases, and vice versa, with the increase in the distance between the layers, the volume and density of the transverse bonds decreases, and in the meantime, the change in the energy of collision particle has had the least effect, and has not had a visible effect on the density of transverse bonds.

### Normal tensile test

**Figure 3:**
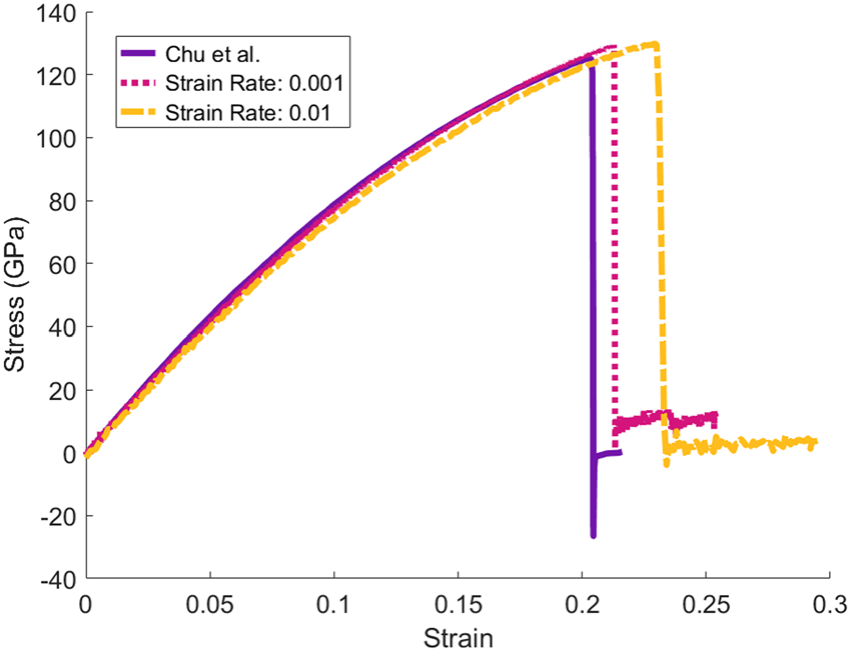
Comparison of SLG strain-stress graphs. The solid line belongs to the reference report^32^. The dotted line is the SLG strain-stress plot at the strain rate of 0.001/ps, and the dashed line is the strain-stress plot of the SLG at the strain rate of 0.01/ps.

In the next step, in order to investigate the strength, each of the structures was separately subjected to a mechanical tensile test. The test was carried out in the direction of the z vector (perpendicular to the graphene sheets) to evaluate the effect of the formation of cross-links between the graphene layers. Before the process, a strain-stress test was conducted in the “zigzag” direction of the single layer graphene (SLG) sheet in order to validate and compare the output chart in this test with a result from a similar study by Chu *et al*. as a reference^32^ (Fig. 3). In the study, the minimum cut-off radius in Airebo potential has been modified by the value of 2 Å, also the strain rate was equal to 0.001/ps, and the time step value was 0.1 fs. In the same conditions, there is a slight difference between the strain-stress plot of the reference test and the chart obtained in this study, this slight difference can be due to the difference in the size of the two graphene. The reference graph refers to graphene with dimensions of 4 × 4 nm, but in the present test, the graphene dimension is 3 × 3 nm.

In all next strain-stress tests, the strain rate was set to 0.01/ps. As shown in Fig. 3, the increase in the strain rate caused a slight displacement in the diagram and increased the failure strain by about 8%. The reason for choosing a higher rate of the strain was increasing the speed of simulations, and reducing the time of the test process. By changing the strain rate from 0.001/ps to 0.01/ps, the simulation speed increases by about 10 times.

**Figure 4:**
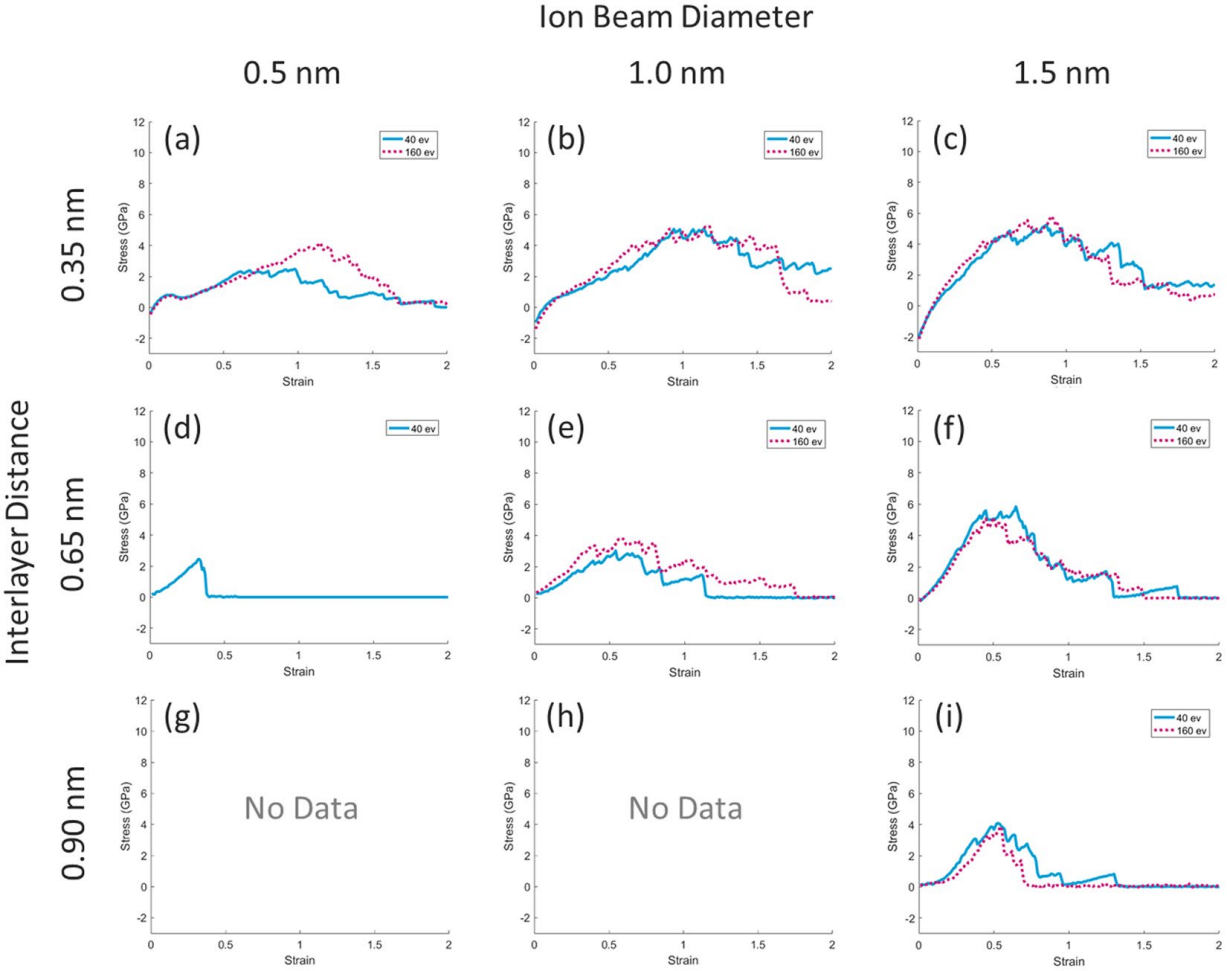
Strain-stress plots of graphene stacks. The blue diagram is related to the configurations generated in the irradiation process at an energy level of 40 eV and the magenta diagram is related to the configurations generated in the irradiation process at an energy level of 160 eV. The top axis represents the diameter of the beam used, and the left axis represents the distance between the graphene layers in the stack. (Each of the graphs results from the average of five strain-stress tests on the five configurations created under equal conditions. Situations that do not meet the requirements for the strain-stress test are displayed as “No Data”).

As previously stated, by changing the irradiation parameters, states are created in which the planes have no connection with each other, or at least there is no bond between the two graphene layers. In this study, all states with at least two non-bonded sheets lacked the necessary requirements of the strain test and therefore no mechanical test was performed on them. These states are depicted in Fig. 4 as “No Data”.

In Fig. 4 it can be seen that as the distance between the graphene layers increases, the ultimate tensile strength in the graphs is reduced, and on the other hand, this value has been increased by increasing the diameter of the collision beam. This quantity has been raised from 4 GPa for a beam with a diameter of 0.5 nm to about 6 GPa for a beam with a diameter of 1.5 nm. The reason for this increase, as seen in the image of the structures formed after irradiation (Fig. 2), is the density of the bonds formed between the layers. In fact, as the diameter of the beam increases, more carbon atoms are separated from the higher layers and transmitted through the interlayer thereby providing the necessary material to create higher density bonds with a longer chain length. The other parameter, ion beam energy, does not have a significant effect on the results, so that in some cases it is seen that the energy of the collision increases the strength and in some cases, in reverse, it reduces the strength. This difference, more than it is related to the beam energy, seems to be due to system fluctuations and collisions and randomized events due to ion collisions on graphene sheets. Although the results were averaging of five separate tests with identical conditions, it should be noted that in the case of (Fig. 4d) at the energy level of 40 electron volts and (Fig. 4i) at the energy level of 160 eV, the results obtained are related to only one configuration and the two configurations respectively which had the necessary conditions for the tensile test. Other configurations have been excluded from the mechanical test due to the lack of links at least between two layers of graphene. It seems that increasing the beam energy at higher interlayer distances, increases the risk of no bond formation so that in the state (d), none of the five configurations obtained after the irradiation process with an energy of 160 eV have had a complete connection. But at the energy level of 40 eV, of five configurations, in one case a complete connection has occurred. Also in the state (i) for 160 eV, of five configurations, only two complete connections have been made, while, in the same conditions for the 40 eV energy level, all five configurations have had a full connection.

**Figure 5:**
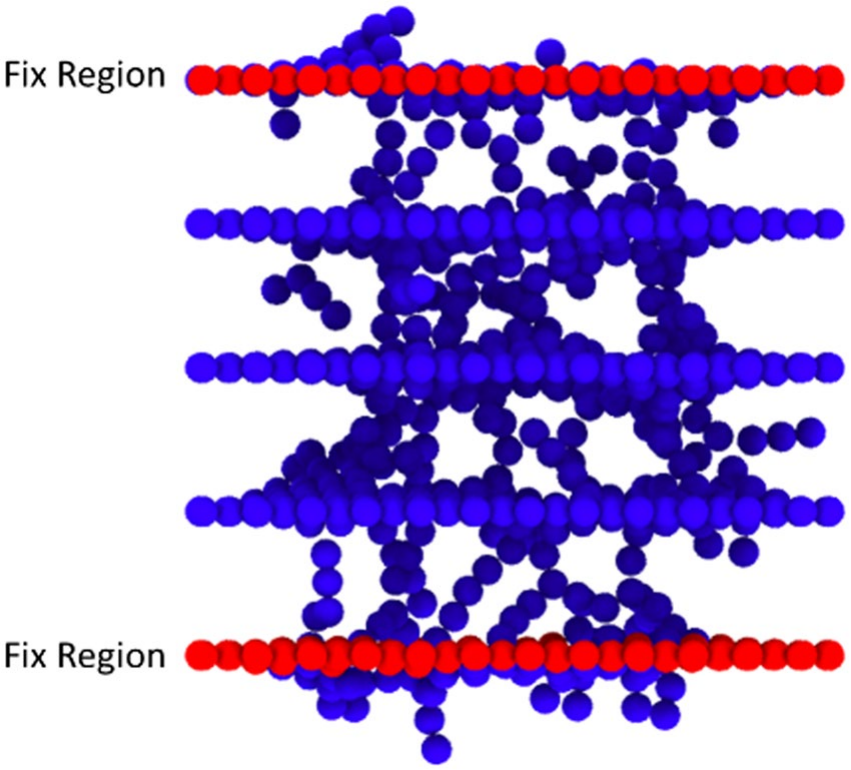
The reason for the negative tension in the stress-strain curves is the compulsion to maintain the upper and lower graphene sheets during the process of relaxation.

As is observable in Fig. 4, the initial value of stress for some cases is negative whereas it should be 0. Prior to performing all stress-strain tests, all structures were relaxed in a constant pressure ensemble for 50 Ps. In addition, to ensure complete relaxation, the energy minimization algorithm, as well as the relaxation of the simulation box, is restored. Such an approach allows both the energy of the atoms at the final configuration to be minimized and the pressure of the system be close to the external pressure (which is zero here). In fact, the reason for the negative tension in the graphs above is the compulsion to maintain the upper and lower graphene sheets during the process of relaxation. This is depicted in Fig. 5 clearly.

### Transverse tensile test

**Figure 6:**
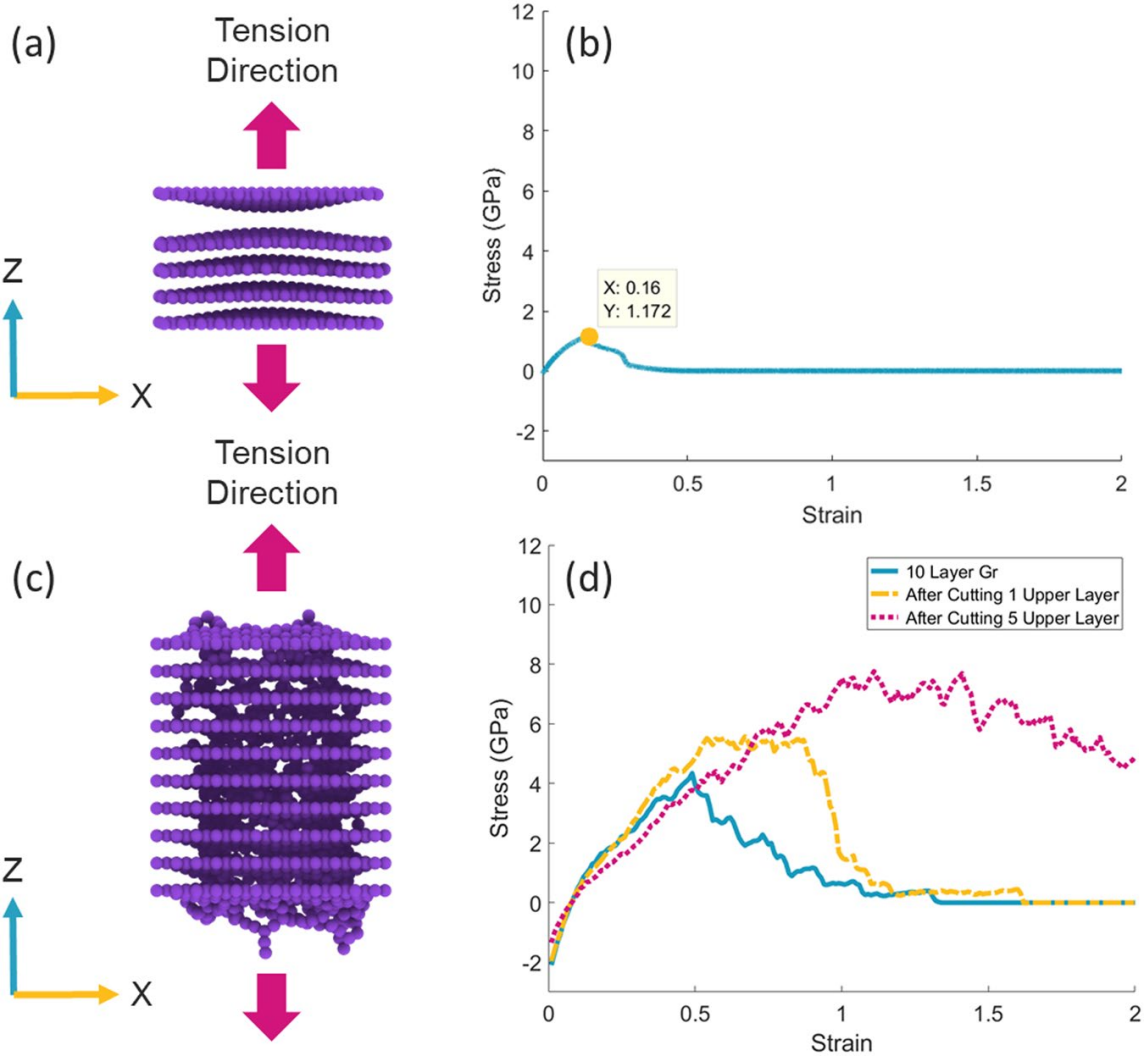
(**a**) Fracture point of interlayer Van der Waals interaction in the multilayer graphene sheet. (**b**) Strain-stress diagram of multilayer graphene sheet when is under loading perpendicular to sheets. (**c**) Graphene stack with 10 layers after the irradiation process and forming covalent bonds. (**d**) Tension test diagram of a 10-layer stack of graphene, the solid line belongs to a 10-layer stack. The dashed line is the result of the tensile test after removal of one upper layer of the stack. The dotted line is the result of strain-stress test after removal of the five upper layers. (All plots are averaged of five strain-stress tests on five different configurations created with equal conditions).

For comparison, two other tensile tests were performed in addition to the previous studies. In the first test, pure multilayer non-exposure graphene was subjected to mechanical testing, in order to compare the physical force between the graphene sheets and the states in which the sheets formed a covalent bond (Fig. 6a). In this case, the number of layers was equal to 5, and the test was performed in the z vector direction (perpendicular to graphene stack) in the same conditions as previous experiments. In this case, the ultimate tensile strength is equal to 1.17 GPa and the two upper layers of the graphene stack are separated in the strain of 0.16 (Fig. 6b). In the second test, the number of graphene layers was considered to be ten, to evaluate the process of the nanopore fabrication and subsequently cross-linking in more number of layers (Fig. 6c). The diameter of the beam in this test was 1.0 nm and the particle energy was considered to be 40 eV. The tensile test was also carried out for the ten-layer graphene in the z-direction vector with the same conditions as before. The ultimate tensile strength for ten-layer graphene was obtained about 4 GPa by averaging five separate tests. As shown in Fig. 6c, the lower layers contain more density of links, the reason it seems to be a transferring of carbon atoms from the upper layers to the lower layers. Indeed, the lower layers receive more carbon atoms from the higher layers, which increases the probability of bonding between the underlying layers. The tensile test was again carried out by removing one and five upper layers of graphene stacks respectively to verify graphene strength in these conditions. As shown in the diagram (Fig. 6d), the removal of the first layer with low bonds density improved the graphene strength by about 40%, also removal of the five upper layers has increased the ultimate tensile strength from about 4 GPa to about 8 GPa, which indicates the more density and strength of bonds in deeper layers of graphene than the surface layers. In addition, we can see that the area of plastic deformation has expanded dramatically by removing the upper layers.

**Figure 7:**
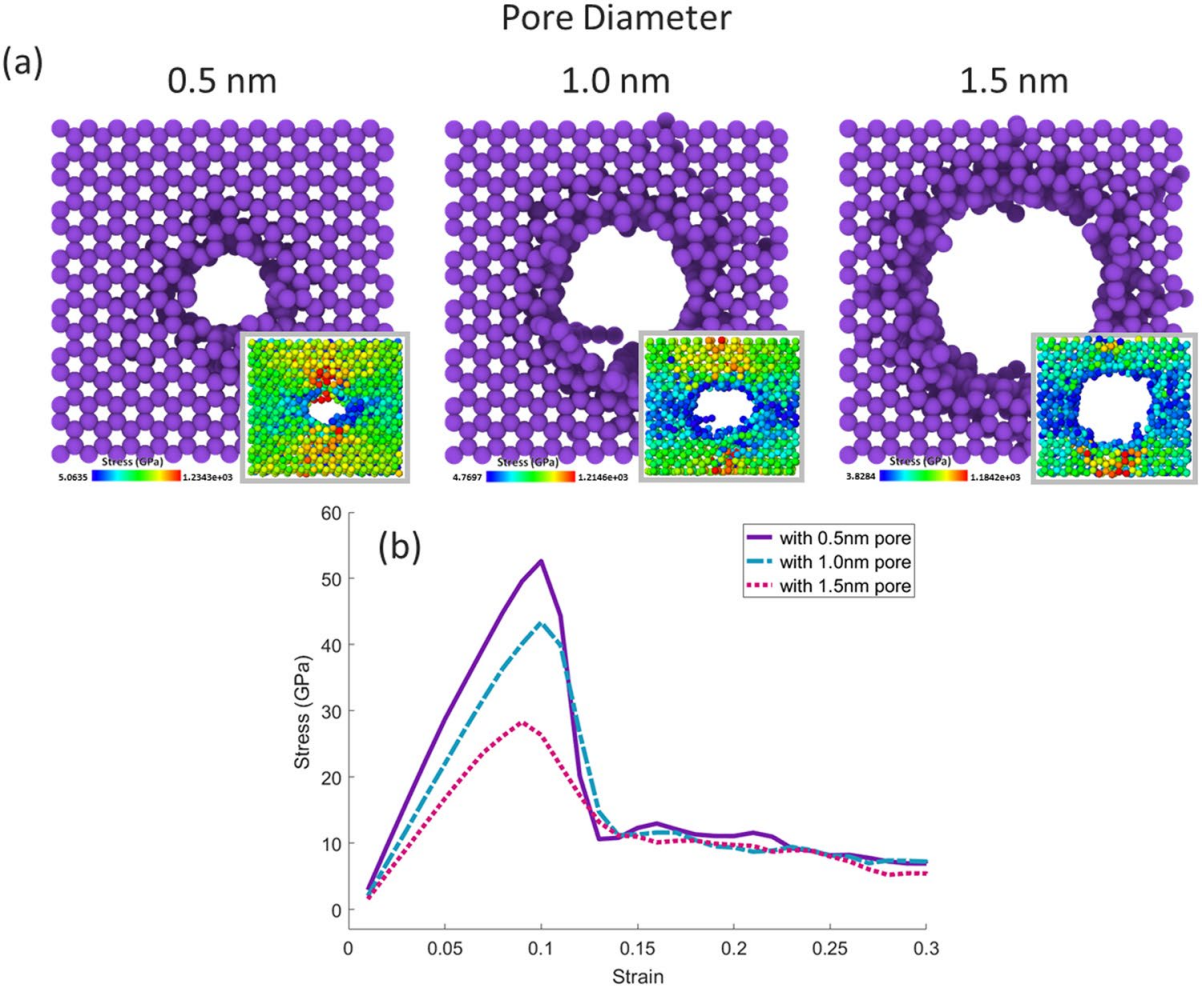
(**a**) Image of pores created with different diameters on a five-layer stack of graphene sheets. (inset) Tension contour image at the point of ultimate tensile strength associated with each configuration. (**b**) The diagram of the strain-stress tests in x vector direction for the configurations shown in (**a**). The solid curve is the test on the graphene stack with a 0.5 nm pore, the dashed curve is the test on the graphene stack with a 1.0 nm pore, and the dotted line is the test on the graphene stack with a 1.5 nm pore.

However, the formation of pores by irradiation process creates bonds between the graphene sheets and reinforces the strength perpendicular to the sheets, in reverse, presence of these pores can weaken the strength in a parallel direction with the sheets. In order to investigate how to change the amount of ultimate tensile strength by changing the size of the pores, three configurations with 0.5 nm, 1.0 nm, and 1.5 nm pore diameter, and a spacing of 0.34 nm, were selected from the configurations obtained during the irradiation process, and The strain-stress test was performed for them in the direction of the x vector “zigzag”. As can be seen in the diagram (Fig. 7), the increase in the size of the pore significantly reduced the graphene strength along the x vector, so that by increasing the size of the pore from a diameter of 0.5 nm to 1.0 nm, about 20% of the graphene strength is reduced, and increasing the diameter of the cavity from 1.0 nm to 1.5 nm resulted in a sharp reduction (about 60%) in the strength. However, in the worst case, graphene with a 1.5-nanometer cavity, the ultimate tensile strength is about 30 GPa, which is far superior to many other materials.

### Nano welding

**Figure 8:**
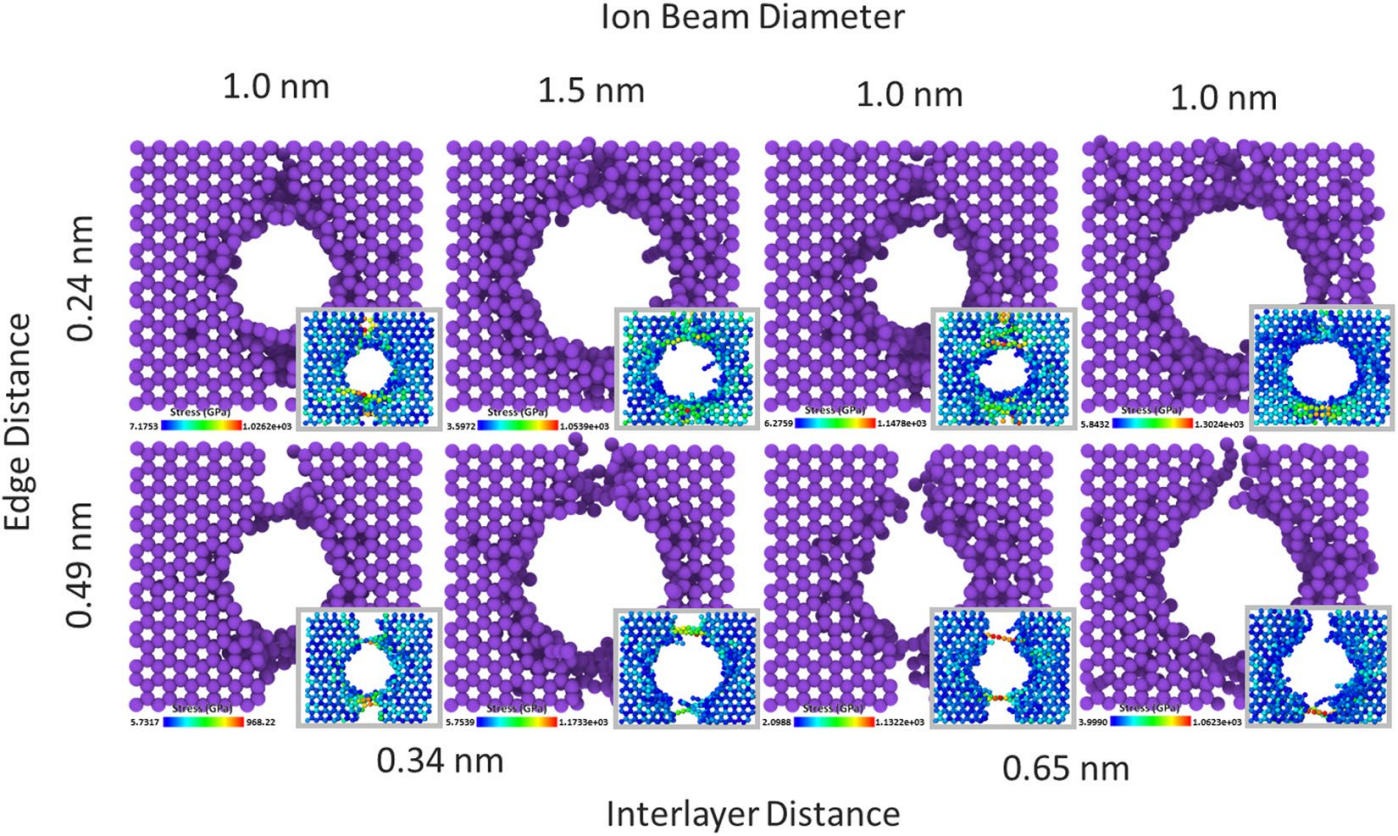
Configurations obtained after the irradiation process in the boundary region between two five-layer stacks of graphene. (inset) Tension contour at the point of ultimate tensile strength.

In addition to all the tests carried out, the irradiation process for joining and strengthening, in which two stacks of graphene were flanked side by side, were also examined. A graphene film contains large boundary regions which are formed by adjoining graphene sheets and stacks. The study of the effect of the ion beam on these areas can also be important. For this purpose, with the same conditions as previous, a similar test was conducted in the boundary areas between the two five-layer stacks of the graphene. Variable parameters in this test are the diameter of the beam, the spacing of the layers and the boundary size between two graphene stacks. The diameter of the beam for this test was 1.0 nm and 1.5 nm. Sheets in the stack were placed at a distance of 0.34 and 0.65 nm to each other, and the size of the boundary regions between the two stacks of graphene were 0.24 nm and 0.49 nm which were created by removing one row and two rows of carbon atoms from the integrated graphene structure, respectively (Fig. 8).

**Figure 9:**
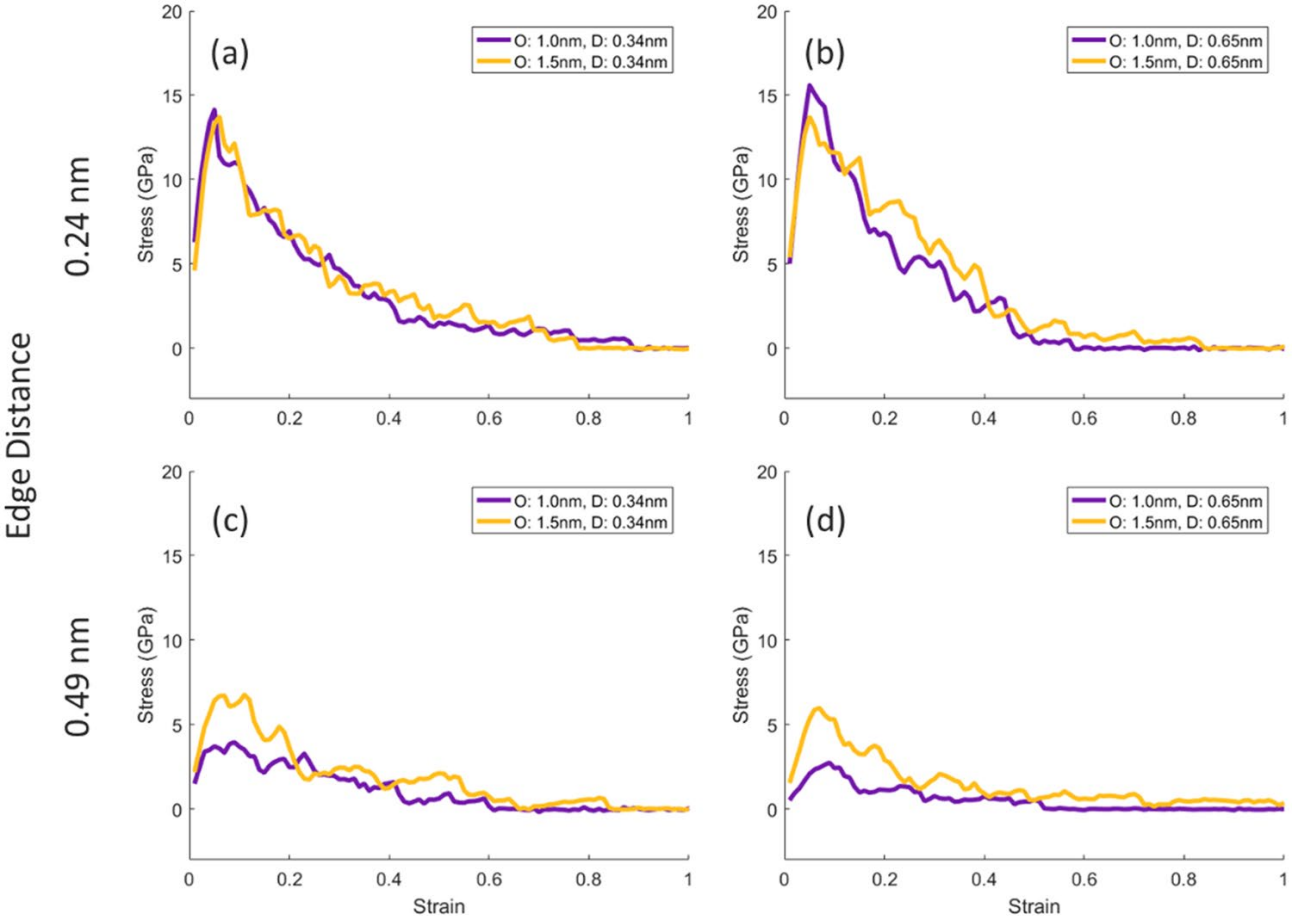
(**a**–**d**) Graphs obtained from the strain-stress test in the direction of x vector “zigzag” for the joined stacks of graphene in the boundary area shown in Fig. 8.

According to the graphs obtained from the strain-stress test on the eight existing states (Fig. 9), increase in the distance between two graphene stacks, namely the size of the boundary area, has the greatest effect on the ultimate tensile strength. By increasing the boundary size from 0.24 nm to 0.49 nm, the ultimate tensile strength has been halved, and this decrease is seen regardless of the distance between the graphene layers and the size of the pore in each of the eight states. Increasing the diameter of the beam from 1.0 nm to 1.5 nm, with a boundary size of 0.24 nm, has not had a significant effect on the graph, but in the case of a 0.49 nm of boundary size, the increase in beam diameter contributed to the increase in strength and left a notable increase in the strain-stress curve. Meanwhile, the change in the distance between the graphene sheets has had the least effect on the ultimate tensile strength. Each of the graphs is averaged over five strain-stress tests on five different configurations created in equal conditions.

**Figure 10:**
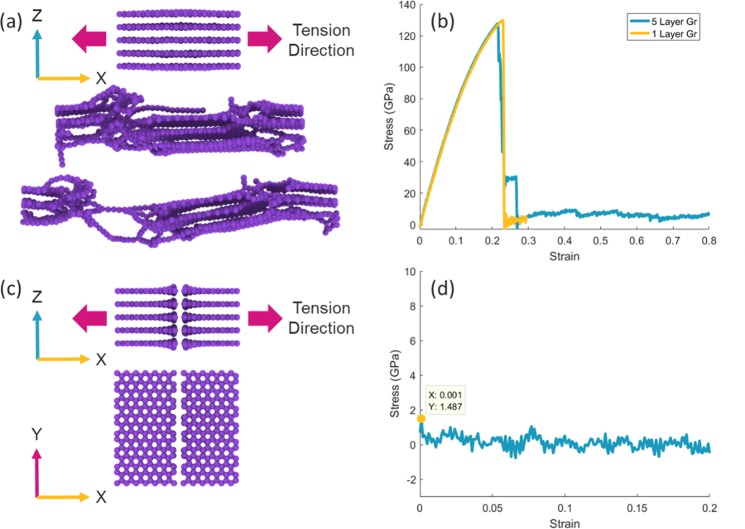
(**a**) A simulation image of a tensile test on a five-layer stack of graphene sheets in a zigzag direction. (**b**) Comparison of strain-stress test on SLG and five-layer graphene stack in a zigzag direction. (The yellow curve is related to SLG and the blue curve is related to the five-layer graphene stack). (**c**) Image of two separate graphene stacks. (**d**) Strain-stress test diagrams for two separate graphene stacks.

For comparison, a pure five-layer graphene stack without porosity was subjected to the tensile test (Fig. 10a). The ultimate tensile strength of this structure was 127 GPa, which is about 1.7% less than the SLG. The amount of the stress in this structure, even after applying a strain of 0.8, has not been reduced to zero, due to the tearing and sliding and the reformation of the bonds between the various layers, which has created a wide area of plastic deformation. In the next, one row of the carbon atoms was removed from the middle of the sheets and then the structure was loaded without performing the irradiation process. In this case, the amount of interaction between two graphene stacks is so small that it cannot be detected by system fluctuations. However, the highest tensile stress recorded is in the strain of 0.001 and it is about 1.5 GPA. Therefore, in the best case, the irradiation process improves the ultimate tensile strength by 1000% in a boundary area between two graphene stacks, which is about one-tenth of the integrated graphene structure. In the worst case, i.e. when the distance between the two stacks is 0.49 Å and the beam diameter is equal to 1 nm, we also see a 2.5 GPa strength in the boundary area after the irradiation process, which is about one-fifth of the ultimate tensile strength in the integrated graphene structure.

## Conclusion

Bonding graphene layers by irradiation process and controlling of the distance between the graphene layers by moisture content, a method for constructing three-dimensional graphene structures was proposed. By presenting this new idea, the following results are achieved:

1. Ion beam energy, have the least effect on the final structure, and the difference between the two structures created by different levels of energy is not tangible. An increase in the diameter from 0.5 nm to 1.5 nm has a significant effect on the increase in the volume of the intermediate links between the graphene sheets.
2. As the distance between the graphene layers increases, the ultimate tensile strength is reduced and increased by increasing the diameter of the collision beam from 4 GPa for a beam with a diameter of 0.5 nm to about 6 GPa for a beam with a diameter of 1.5 nm.
3. Performing a transverse tensile test after ion beam irradiation on a ten-layer graphene stack, for all simulations, the lower layers contain more density of links, because of transferring of carbon atoms from the upper layers to the lower layers. Indeed, the lower layers receive more carbon atoms from the higher layers, which increases the probability of bonding between the underlying layers. The removal of the first layer with low bonds density improved the graphene strength by about 40%. Also, removal of the five upper layers has increased the ultimate tensile strength from about 4 GPa to about 8 GPa, which indicates the more density and strength of bonds in deeper layers of graphene than the surface layers.
4. Increasing the size of the pores significantly has reduced the graphene strength so that by increasing the pore size from 0.5 nm to 1.0 nm, about 20% of the graphene strength is reduced, and increasing it from 1.0 nm to 1.5 nm resulted in a strong reduction (about 60%) in the strength. However, in the worst case, i.e. porous graphene with a 1.5-nanometer pore, the ultimate tensile strength is about 30 GPa, which is far superior to many other materials.
5. Finally, nano welding of graphene sheets was proposed via ion beam irradiation. By increasing the boundary size from 0.24 nm to 0.49 nm, the ultimate tensile strength has been halved. Increasing the diameter of the beam from 1.0 nm to 1.5 nm, with a boundary size of 0.24 nm, has not had a significant effect on the graph, but in the case of a 0.49 nm of boundary size, the increase in beam diameter contributed to the increase in strength and left a notable increase in the strain-stress curve. Meanwhile, the change in the distance between the graphene sheets has had the least effect on the ultimate tensile strength. As a fantastic result, in the best case, the irradiation process improves the ultimate tensile strength by 1000% in the boundary area between two graphene stacks, which is about one-tenth of the integrated graphene structure.

## Supplementary information

Graphene Nanopore Creation
